# Trans-Sclera Electrical Stimulation Improves Retinal Function in a Mouse Model of Retinitis Pigmentosa

**DOI:** 10.3390/life12111917

**Published:** 2022-11-17

**Authors:** Feng Liu, Mengrong Zhang, Guoyin Xiong, Xiu Han, Vincent Wing Hong Lee, Kwok-Fai So, Kin Chiu, Ying Xu

**Affiliations:** 1 Guangdong-Hongkong-Macau Institute of CNS Regeneration, Key Laboratory of CNS Regeneration (Ministry of Education), Jinan University, 601 West Huangpu Ave., Guangzhou 510632, China; 2 State Key Laboratory of Ophthalmology, Zhongshan Ophthalmic Center, Sun Yat-sen University, Guangdong Provincial Key Laboratory of Ophthalmology and Visual Science, Guangzhou 510060, China; 3 Department of Ophthalmology, The University of Hong Kong, Hong Kong SAR, China; 4 Co-Innovation Center of Neuroregeneration, Nantong University, Nantong 226019, China; 5 The State Key Laboratory of Brain and Cognitive Sciences, The University of Hong Kong, Hong Kong SAR, China; 6 Department of Psychology, The University of Hong Kong, Hong Kong SAR, China

**Keywords:** retinitis pigmentosa, photoreceptor degeneration, multi-electrode array, retinal ganglion cells, electric stimulation

## Abstract

Retinitis pigmentosa (RP) is a photoreceptor-degenerating disease with no effective treatment. Trans-corneal electrical stimulation has neuroprotective effects in degenerating retinas, but repeated applications cause corneal injury. To avoid the risk of corneal damage, here we tested whether repetitive trans-sclera electrical stimulation (TsES) protects degenerating retinas in rd10 mice, a model of RP. At postnatal day 20 (P20), the right eyes of rd10 mice were exposed to 30 min of TsES daily or every other day till P25, at the amplitude of 50 or 100 μA, with zero current as the sham. Immunostaining, multi-electrode-array (MEA) recording, and a black-and-white transition box were applied to examine the morphological and functional changes of the treated retina. Functionally, TsES modified the retinal light responses. It also reduced the high spontaneous firing of retinal ganglion cells. TsES at 100 μA but not 50 μA increased the light sensitivities of ganglion cells as well as their signal-to-noise ratios. TsES at 100 μA increased the survival of photoreceptors without improving the visual behavior of rd10 mice. Our data suggest that repetitive TsES improves the retinal function of rd10 mice at the early degenerating stage, therefore, it might be an effective long-term strategy to delay retinal degeneration in RP patients.

## 1. Introduction

Retinitis pigmentosa (RP) is a group of inherited retinal degenerative diseases characterized by gradual losses of rods and cones, eventually leading to blindness [1]. Although it affects 1 in 4000 people worldwide, there is no effective treatment so far. One reason that hinders the treatment effect is the complexity of the RP pathogenesis. First, a large number of genetic defects are associated with RP, including mutations affecting the phototransduction cascade, visual cycle, ciliary structure and transport, and the interphotoreceptor matrix [2]. Each of these genes corresponds to a gene-specific subtype of RP with a specific age of onset, visual impairment, retinal structure, and rate of progression, which therefore require different treatment strategies. Second, multiple pathological changes happen during retinal degeneration, which include oxidative stress [3], inflammatory responses [4], vascular dysfunction [5], and so on. Strategies targeting more than one pathogenesis may be needed to treat RP effectively. Currently, many strategies have been applied to protect or replace photoreceptors in RP, including neurotropic factors, anti-inflammatory or antioxidant agents, stem cell therapy, gene therapy, retinal implants and prosthesis, and electric stimulation (ES) [6]. Based on its non-invasive property and broad range of biomedical effects on both biomolecules and cells [7], ES has been explored to treat eye diseases in the past two decades with promising therapeutic effects.

ES induces vision protection in both animal and clinical studies. In clinical studies, it is applied to treat patients with optic neuropathy, glaucoma, RP, age-related macular degeneration (AMD), and retinal artery occlusion [8]. ES was also used in animal experiments to treat various animal models with photoreceptors or retinal ganglion cell (RGC) degeneration [8]. By delivering microcurrent to the target tissue, ES can interact with cellular components and directly impose various biochemical effects on the cells, such as disrupting extracellular structured water, controlling electroosmotic fluid flow, opening voltage-gated channels, imposing mechanical forces on the tension sensitive components etc. These effects enable ES to interfere with multiple pathological processes that may protect retina in eye diseases.

However, clinical trials failed to establish strong and conclusive supports for the therapeutic efficacy of ES on retinal diseases. One of the major reasons is the lack of an optimized and standard stimulation protocol [7]. The method to deliver the current to the eyeball is suggested to be crucial for the treatment outcome by affecting the distribution of the current in the eyeball [8]. Defined by the location of the electrodes, the electric current is delivered to the eye through several routes, including trans-corneal ES (TcES), transpalpebral ES, transdermal ES, etc. Among all the routes, TcES is the most used method to treat RP in clinics [8], and is effective to protect photoreceptors in various animal models, including RCS rats [9], light-induced [10] or MNU-induced photoreceptor degeneration rats or mice [11], rhodopsin P347L transgenic rabbits [12], P23H-1 rats [13] and rd10 mice [14]. However, repeated applications cause injury to the cornea and hinder the long-term treatment of RP, and cannot be applied to patients with corneal diseases [15]. To protect the retinal neurons while leaving the cornea intact, we adopted a new way to achieve electrical stimulation named trans-sclera electric stimulation (TsES). In this study, we explored whether repetitive trans-sclera electrical stimulation (TsES) may protect the degenerated retina in rd10 mice, a model of RP [16].

## 2. Materials and Methods

### 2.1. Animals and Treatment

Retinitis pigmentosa animal model rd10 mice (B6.CXB1-Pde6b^rd10^, RRID: IMSR_JAX:004297) were purchased from Jackson Laboratory (Bar Harbor, ME, USA). Animals of either sex were maintained under standard laboratory conditions with 12 h light/dark cycle and unlimited access to food and water. All animal-related procedures were performed following the Statement of the Association for the Use of Animals in Ophthalmic and Visual Research and were approved by the Qualified Ethics Committee of Jinan University. The number of animals used, and their suffering, was minimized by all efforts.

**Figure 1:**
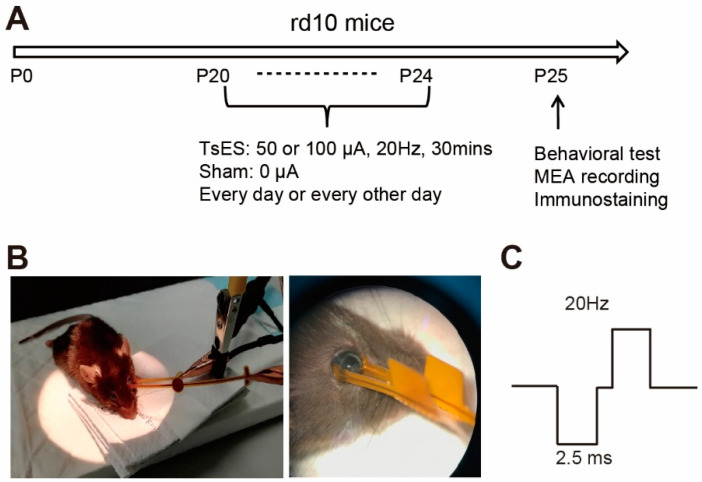
Experimental protocol and setup of electric stimuli. (**A**) Experimental protocol. The rd10 mice were treated with trans-sclera electric stimuli (TsES) for 30 min every day or every other day from the postnatal day (P20) till P24, then animals were tested with behavior and retinal extracted for multi-electrode-array (MEA) recording or immunostaining. For the treatment, the current of TsES was 50 or 100 μA, and for the sham group, no current (i.e., 0 μA) was applied. (**B**) Illustration of the pair of pads on the sclera of a mouse, which delivers the electric current. (**C**) Protocol of the electric current.

Animals were randomly divided into two groups including the trans-sclera electric stimulation (TsES) treated group and the sham control. As photoreceptors start to degenerate around postnatal day 17 (P17), we started the TsES treatment since P20 [16]. The TsES stimuli were applied on the left eye of rd10 mice every day or every other day for 30 min till P24. Each time before the TsES application, animals were anesthetized with tribromoethanol (0.14 mL/10 g body weight of 1.25% solution). Then at P25, when rod degeneration reaches its peak, retinas were extracted and either recorded by multi-electrode-array (MEA) to get the light responses or fixed for immunostaining (protocol see Figure 1A). Behavioral test was also carried out to by a black-white-transition box which test the ability of the animal to tell luminance.

### 2.2. Application of TsES

To apply the TsES, a self-developed multi-channel ES generator was utilized as previously described [17]. The generator utilized a microcontroller unit (MCU) command that enable the simultaneous application of multiple ES parameters with an interactive touch screen. It consists of a six-channel current digital to analog converter as the source of ES and each channel has an independent current output. Then one of the output channels was connected to a pair of gold pads that positioned on the temple and nasal sclera surface of the left eye of a rd10 mouse as illustrated in Figure 1B. The ground line was connected to normal household electric devices as ground reference. Based on reported protocols [18], bi-phasic electric pulse (square wave, 2.5 ms pulse width, 1 ms inter-pulse-interval) was applied at the frequency of 20 Hz for 30 min, with amplitude of either 0, 50 or 100 μA, with 0 μA served as the sham control (Figure 1C). Then Erythromycin Eye Ointment was applied on the stimulated eyes, and animals were returned to the cage after recovery.

### 2.3. Visual Behavior Test

The ability of mice to tell luminance was tested by a black-white transition system device (custom-made by Metronet Technology Ltd., Hong Kong SAR, China) as we described before [19]. The system contains an illuminated white box and a black box of the same size (16 cm × 16 cm × 25 cm) separated by a door that allows the mouse to move freely. Two infrared cameras mounted on top of each box recorded the movement of the animal inside the boxes. The recorded video was analyzed by Etho Vision XT 8.0 analyzing Software (Noldus Information Technology BV, Wageningen, The Netherlands) to calculate the time of mice staying in the black box during the 5 min test.

### 2.4. Multi-Electrode-Array Recording and Data Analysis

To examine the light-evoked activities of retinal neurons, MEA recording from ex vivo retina was performed on whole-mount retinas as previously described [19]. Briefly, after 3 h dark adaptation and under dim red light, retinas were isolated from retinal pigment epithelium and underlying choroid and sclera. A small region of the retina (about 2 × 2 mm^2^) from the middle area was placed tightly on an MEA array with ganglion cells facing the electrodes. The array consists of 8 × 8 electrodes of 20 μm in diameter spaced 100 μm apart (P210A, Alpha MED Scientific Inc., Osaka, Japan). Then the MEA array with the retina was transferred to the recording stage, connected to a MED 64 amplifier (Alpha MED Scientific Inc., Osaka, Japan), and continuously perfused with oxygenated AMES’s medium (8.8 g/L, Cat#A1420, Sigma Aldrich, Shanghai, China) containing NaHCO_3_ (1.9 g/L, Cat#792519, Sigma Aldrich, Shanghai, China). The bath solution flowed at a rate of ~4 mL/min and the temperature maintained at 31–33 °C. The full-field light stimuli were presented by a white light-emitting diode (LEDW7E; Thorlabs, Newton, NJ, USA) controlled by the MED 64 amplifier, and the protocol included a 30 times repetition of 10s duration with light ON for the first 2s. The flash intensity applied included 4.20, 5.46, 5.96, 6.68, 7.09, and 7.67 log photons/μm^2^/s.

Data were acquired by Mobius software (Alpha MED Scientific Inc., Osaka, Japan), with ample rate at 20 kHz and band-pass filtered between 1 and 5000 Hz. The waveform of microERG was extracted first by applying a 100 Hz low-pass filter on the raw waveforms recorded under flash intensity of 7.67 log photons/μm^2^/s [19,20]. Our recent work has identified the cellular origins of the first large positive peak of microERG (P1) and the second negative peak (N2) during light ON as ON bipolar cell and photoreceptors respectively [19]. Furthermore, there were also two peaks after light OFF which may arise from OFF bipolar cells. Thus, to evaluate the light responses from photoreceptors and inner retinal neurons, the amplitude of each peak was measured from the baseline to peak for each channel, averaged and compared among animal groups.

To separate the spikes of retinal ganglion cells (RGCs), a 100 Hz high-pass filter was applied to the raw waveforms, and those multi-unit spikes were separated into single-unit spike trains using Offline Sorter software (Version 3.3.5; Plexon, Dallas, TX, USA). Then the sorted data were exported to Spike 2 software (Version 8; CED, Cambridge, UK), and further processed with Matlab software (Version 2022a; MathWorks, Natick, MA, USA) and R language (version 3.5.1; The R Foundation, New Zealand). The peristimulus time histogram (PSTH) and corresponding raster plot with a 10 ms bin width were displayed and analyzed for the activities of each unit. Light responses were calculated by subtracting the spontaneous firing rate (average response before light onset) from the peak or average spike firing rate during the first 2 s of light onset (ON response) or offset (OFF response) or the average of both ON and OFF (ON-OFF response). To assess signal transmission speed, the latency was calculated as the interval between the light onset (or offset) and the peak of PSTH. The signal-to-noise ratio (SNR) was further calculated by dividing the light response of each ganglion cell by the standard deviation of its spontaneous firing, to evaluate the efficiency of visual information transmission in the retina [19,21]. To further assess the sensitivity (σ) of the light responses, the Hill equation was used to fit the light-intensity curve, and σ was computed as the light intensity resulting in the half-maximum response [19,22]. A lower σ value suggests higher light sensitivity.

### 2.5. Tissue Processing, Immunocytochemistry, and Image Processing

To examine the retinal structure, the eyeballs were removed after sacrificing the animal and fixed in 4% paraformaldehyde for 30min. The eyes were then rinsed with PBS (0.01 M, pH 7.4), cryoprotected in PBS solution containing 30% sucrose overnight at 4 °C, and embedded in the optimal cutting temperature compound (OCT; Tissue Tek, Torrance, CA, USA). Retinas were then cryo-sectioned through the optic disk longitudinally at a thickness of 15 μm using a Leica microtome (CM 1950, Leica Biosystems, Nussloch, Germany), and sections were transferred and mounted on glass slides for future processing. For DAPI staining, retinal sections were incubated with 4′,6-diamidino-2-phenylindole (DAPI; 1:2000, Sigma-Aldrich, Beijing, China, RRID: AB_2307445) at room temperature for 5 min before mounting and sealed.

Fluorescent images were captured with a fluorescence microscope (Zeiss LSM700, Carl Zeiss, Gottingen, Germany). As photoreceptors of rd10 mice deteriorate from the center to the periphery, we evaluated photoreceptor survival from the center to the peripheral region, by measuring the thickness of the outer nuclear layer (ONL) at 400 μm intervals beginning from the optic nerve head center. For each image, the measurement was done by drawing three lines vertically across the retinal section at the left, middle, and right locations. The length of lines was measured by Image J software (NIH, Bethesda, MD, USA) and averaged to get the number for one image. For each retina, values from 2–4 images were averaged to create one data point, and the mean value for a group was calculated by averaging all the data points from the retinas within that group.

### 2.6. Statistical Analysis

All data were presented as mean ± SEM. Statistical analysis was performed using Prism 7 software (GraphPad Software, San Diego, CA, USA). The Shapiro–Wilk test was applied to assess the normality of data. Statistical significance was analyzed with Student’s t-test or one-way ANOVA (followed by Tukey’s post-hoc tests) for parametric data, and Mann Whitney test or Kruskal–Wallis (followed by Dunn’s post-hoc tests) for non-parametric data. *p* value < 0.05 was considered statistically significant. The “n” indicates the total number of mice examined for each group unless otherwise stated.

## 3. Results

Compared to the contralateral control eye, there were no changes on the transparent cornea. At the site where pad inserted, no tissue reactions such as hemorrhage and edema were observed. The overall ocular condition of the treatment eye was comparable to the contralateral eye.

### 3.1. TsES at 100 μA Modified the Retinal Light Responses

**Figure 2:**
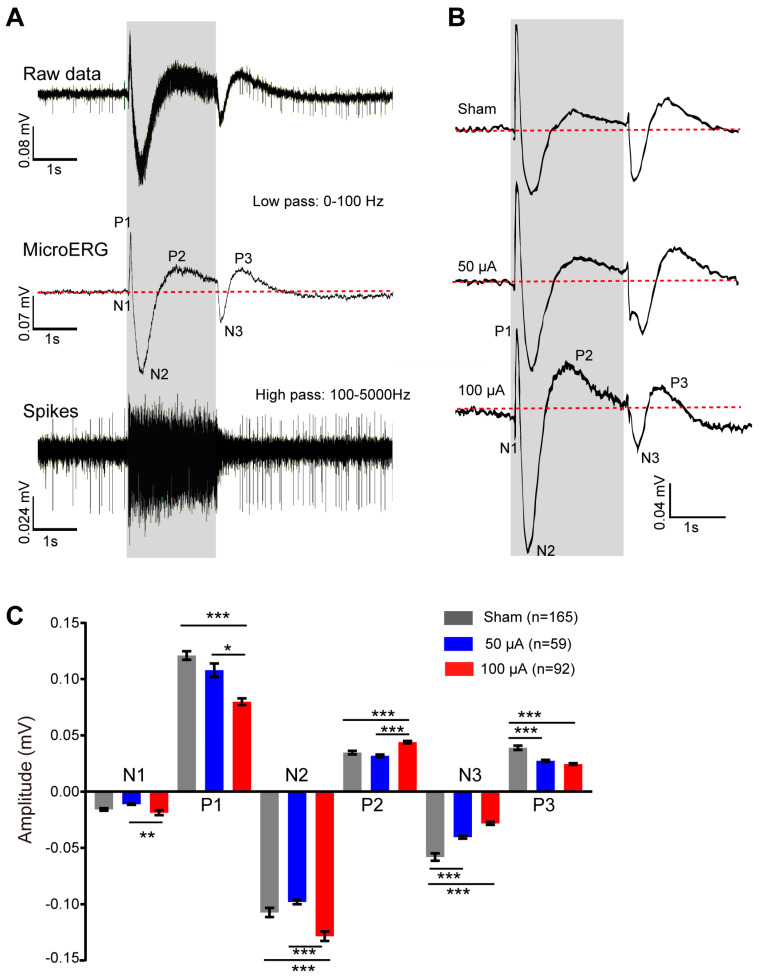
TsES modified the retinal light responses in rd10 mice. (**A**) Isolation of the light responses of photoreceptor and inner retinal cells from microERG trace recorded by MEA from a WT retina. Raw data of MEA is composed of a fast spiking from retinal ganglion cells on top of slow potential changes of microERG arising from the photoreceptor and inner retinal neurons. Applying a low pass filter isolated the microERG traces with a series of negative and positive peaks arising from photoreceptors and inner retinal neurons. Applying a high-pass filter removed the microERG components, leaving only spikes from RGCs. The gray area indicates 2s light stimuli at the intensity of 7.67 log photons/μm^2^/s. (**B**) Example traces of microERG from a channel recorded from the retinas of different animal groups. Red lines in microERG traces mark the baseline from which the amplitude of each peak was measured. (**C**) Average amplitude of all the peaks for different groups. TsES modified many of the peaks. *, *p* < 0.05, **, *p* < 0.01; ***, *p* < 0.001, one-way ANOVA. Numbers indicates the number of channels recorded by MEA from three sham-treated rd10, two 50, and two 100 μA treated animals. Data are expressed as mean ± SEM.

To evaluate the functional improvement, we applied multi-electrode-array (MEA) recording to analyze the light response of photoreceptors, bipolar cells, and ganglion cells in rd10 mice. The light responses of photoreceptor and inner retinal neurons were extracted from microERG waveforms after low-pass filtering the MEA data [20,23]. A tiny sharp negative peak (N1) followed with a large sharp positive peak (P1) then a negative potential (N2) and a slow positive potential (P2) appeared after light onset (Figure 2A for WT mouse and B for rd10 mice). After light offset, a sharp negative peak (N3) followed by a slow rising potential (P3) were also observed. Therefore we analyzed the amplitude of all waves in the microERG waveforms. We firstly analyzed the MEA data and found no difference between the data from TsES applied daily and every other day, so, for simplification, we combined the data and compared the out-coming of TsES at different currents. Data showed that TsES at both currents significantly decreased the amplitudes of N3 and P3 waves which related to OFF bipolar cells (*p* < 0.001 vs. sham). At 50 μA, TsES hardly affected the amplitude of N1, P1, N2 and P2 waves compared with the sham group. In contrast, TsES at 100 μA slightly increased the N1 amplitude to 118% of the sham (*p* = 0.32) and significantly enhanced the amplitudes of N2 wave to 120% of the sham (*p* < 0.001) and P2 wave to 127% of the sham (*p* < 0.001) (Figure 2C). As the N1 and N2 waves arise from photoreceptors [19,20] and P1, P3 waves arise from ON and OFF bipolar cells respectively, TsES at 100 μA therefore modulated the light responses of retinal neurons, including photoreceptors, ON and OFF bipolar cells. Specifically, it improved the light responses of photoreceptors of rd10 mice.

### 3.2. TsES at 100 μA Enhances the Light Function of RGCs

As TsES improved the light response of photoreceptors but not ON bipolar cells, we next explored how TsES affected the response of retinal ganglion cells (RGCs). Spikes of RGCs were separated by high-pass filtering the MEA data (Figure 2A) and sorted the spikes for each individual cell. In the rd10 retina, with photoreceptor degenerates, light response decrease and spontaneous firing increase abnormally, which hampers the reliability of signal transmission [24]. Therefore, we analyzed the spontaneous firing, light response (including peak and average responses), and Signal-to-Noise ratio (SNR) of rd10 RGCs at the saturating flash intensity first.

**Figure 3:**
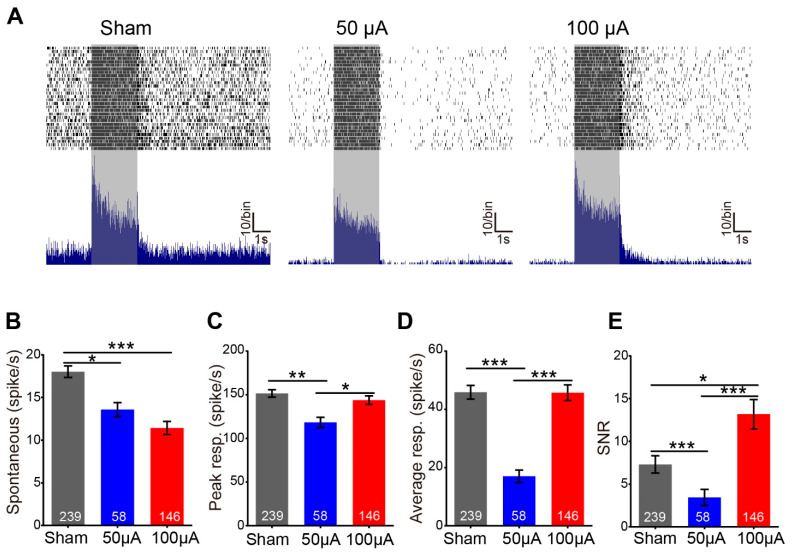
TsES at 100 μA increased the light responses of retinal ganglion cells in rd10 mice. (**A**) PSTH (bottom) and corresponding raster plots (top) of representative RGCs recorded in different groups. The duration of the light stimulus is indicated by the gray area with a duration of 2s and intensity of 7.67 log photons/μm^2^/s. In all groups, RGC responded well to the light stimuli, and TsES decreased the high spontaneous firing in rd10 mice. (**B–E**) Mean spontaneous firing (**B**), peak response (**C**), average response (**D**), and signal-to-noise ratio (SNR) (**E**) for different groups. TsES at 100 μA significantly decreased the spontaneous firing and increased the SNR in RGCs of rd10 mice. TsES at 50 μA decreased the spontaneous firing, but also the light response and SNR of rd10 RGCs. *, *p* < 0.05; **, *p* < 0.01; ***, *p* < 0.001, one-way ANOVA. The numbers in the bar indicate the number of cells recorded by MEA. Data are expressed as mean ± SEM.

Compared to the sham group, TsES significantly decreased the spontaneous firing of rd10 RGCs from 18 ± 0.7 to 13.6 ± 0.8 spikes/s for 50 μA and 11.4 ± 0.7 spikes/s for 100 μA (Figure 3A,B). TsES at 50 μA did not improve the light responses, instead, it decreased the peak and average light response of rd10 RGCs as well as the SNR (Figure 3C–E). In contrast, TsES at 100 μA increased the light response to the level of the sham group (Figure 3C,D, *p* < 0.05 vs. 50 μA), it also significantly improved the SNR to 1.8 fold of the sham group (*p* < 0.05, Figure 3E). Therefore, TsES at 100 μA enhanced the light function of rd10 RGCs by decreasing the spontaneous firing and improving the SNR.

**Figure 4:**
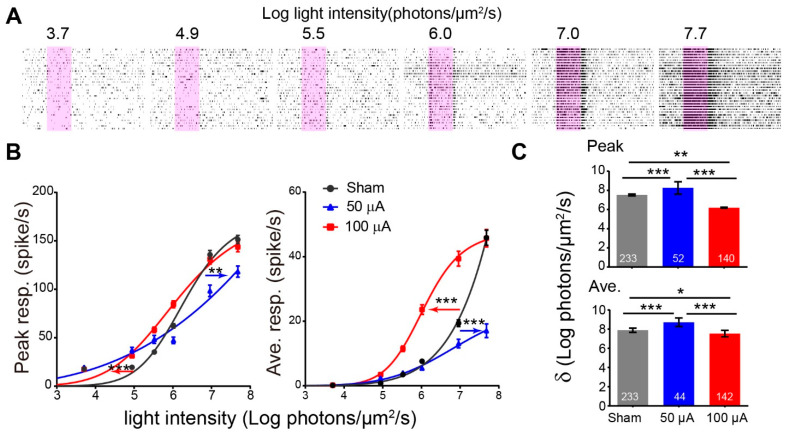
TsES at 100 μA increased the light sensitivity of rd10 ganglion cells. (**A**) Raster plot of an example cell from sham-treated rd10 to light flashes (pink regions) with increasing intensities (values shown above the stimuli, with the unit of log photons/μm^2^/s). (**B**) Peak response (left) or average response (right) versus light-intensity curves for three groups. TsES at 50 μA shifted the curve to the right and TsES at 100 μA shifted the curve to the left compared with the sham-treated rd10. (**C**) Average value of σ, the flash intensity that evokes half-maximum response for peak (top) and average response (bottom). TsES at 100 μA significantly decreased the value of σ from the sham group. *, *p* < 0.05, **, *p* < 0.01; ***, *p* < 0.001, two-way ANOVA for (**B**) and one-way ANOVA for (**C**) Data are expressed as mean ± SEM.

The functional improvement of rd10 RGCs by TsES was further proved by the increase of photosensitivity in rd10 RGCs with increasing flash intensities ranging from 3.7 to 7.7 log photons/μm^2^/s. As shown in Figure 4A, the frequency of spikes firing increased with the increasing flash intensity. Compared with the sham group, the light response curve was shifted rightward by TsES at 50 μA indicating a decrease of light sensitivity (Figure 4B). This change was reversed by the 100 μA condition with the leftward shift. Calculating σ (the light intensity that elicits the half-maximum response) showed that σ of both the peak response and the average response was increased after TsES at 50 μA (*p* < 0.001 for both peak and average response), indicating a decrease of light sensitivity. In contrast, TsES at 100 μA significantly decreased the value of σ for both peak and average response (peak: *p* < 0.01 vs. sham; average: *p* < 0.05 vs. sham) (Figure 4C). Therefore, TsES at 100 μA enhanced the light sensitivity of rd10 RGCs.

### 3.3. TsES Hardly Changes the Visual Behavior of rd10 Mice

**Figure 5:**
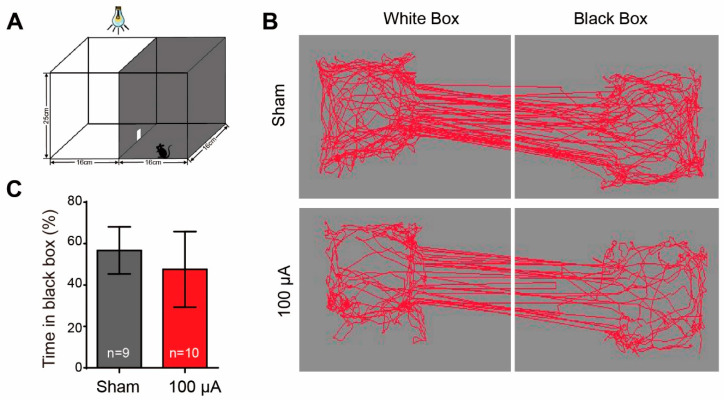
TsES at 100 μA hardly improved the visual behavior of rd10 mice. (**A**) Illustration of the black/white transition box for the visual behavior test. (**B**) Moving tracks (red lines) of animals in the black and white boxes. (**C**) Percentage of time spent in the black box to the total time spent in both boxes for different animal groups. TsES at 100 μA hardly changed the time of rd10 mice staying in the black box. Data are expressed as mean ± SEM.

After identifying the functional improvement of RGCs by TsES at 100 μA, we next evaluated whether the treatment may improve the visual behavior of rd10 mice by a black-white-transition box (Figure 5A). To our disappointment, rd10 mice spent similar time in the dark chamber (<60% of total duration) with or without TsES (Figure 5). Therefore, TsES hardly improved the ability to detect luminance in rd10 mice.

### 3.4. TsES Improves the Survival of rd10 Photoreceptors

**Figure 6:**
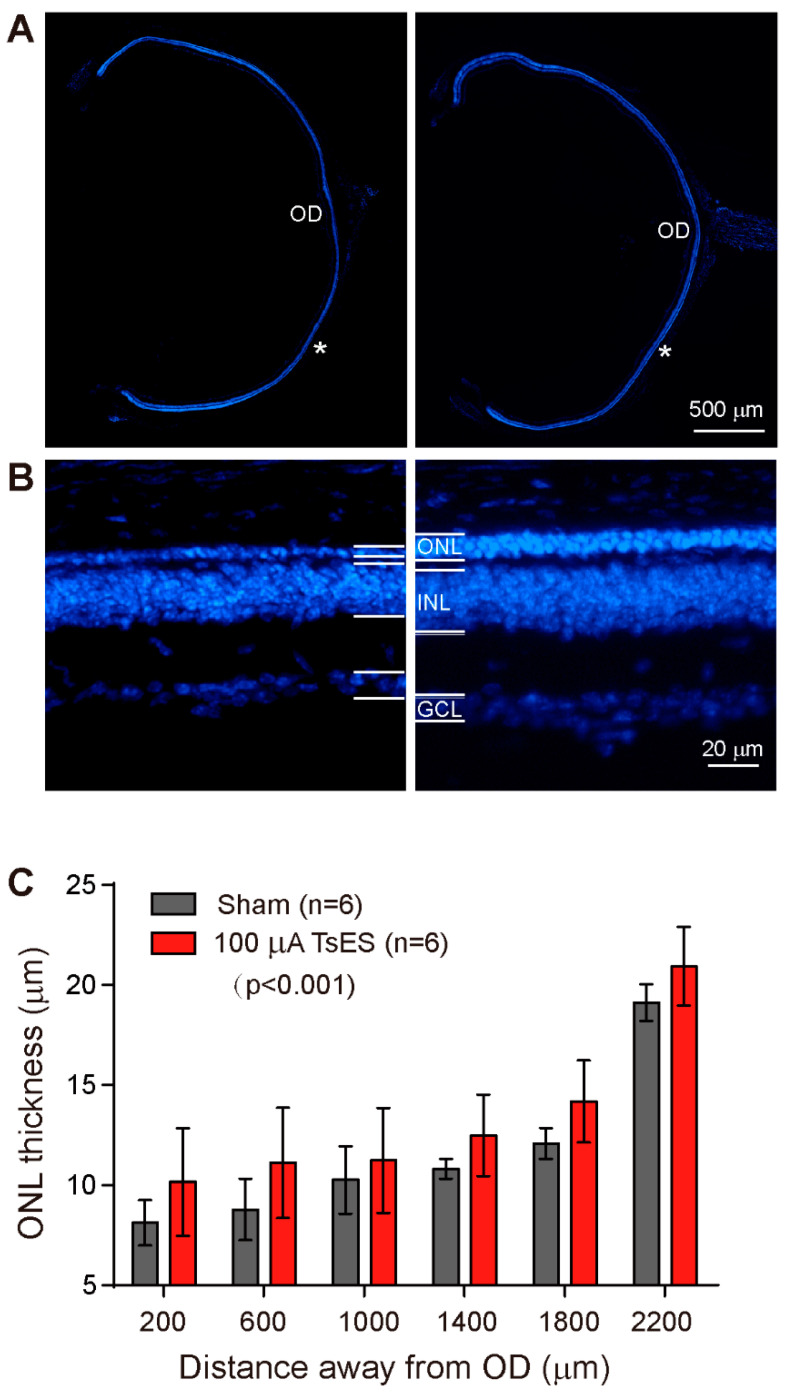
TsES at 100 μA increased the survival of rd10 photoreceptors. (**A**) Ocular cup images covering the full size of the retina with the sampled region (*) at 1 mm away from the center of the optic disk (OD) enlarged and shown in (**B**). Retinal sections were stained with DAPI. The outer nuclei layer (ONL) was thin in rd10 mice and TsES slightly increased the thickness of the rd10 retina. (**C**) The ONL thickness from the center to the peripheral region of rd10 mice. TsES at 100 μA slightly but significantly increased the ONL thickness of the rd10 retina. ONL, outer nuclei layer; INL, inner nuclei layer; GCL, ganglion cell layer. Data are expressed as mean ± SEM. One-way ANOVA with repetitive measurement was applied for the statistical comparison.

With the improvement of RGC function but not visual behavior, we finally examined the survivor of photoreceptors by immunostaining retinal sections with DAPI and measuring the thickness of ONL where somas of photoreceptors are located. In rd10 mice, the ONL layer was thin with only one layer of soma remaining, TsES at 100 μA increased the number of layers to 2–3 layers (Figure 6A,B). The thickness of ONL from the center to the peripheral region (shown as distance away from the optic disk center) was slightly higher in TsES treated group than the sham group at each location, with a significant difference (*p* < 0.001) as a whole group (Figure 6C). Therefore, TsES improved the survival of rd10 photoreceptors.

## 4. Discussion

### 4.1. TsES Protects the Degenerated Retina

Our current study applied a new strategy of applying electric stimuli on the sclera instead of the corneal of diseased eyes and investigated its effect on the rd10 retina. We found that TsES at 100 μA improved the light responses of photoreceptors and RGCs, and it also increased the survival rate of photoreceptors in rd10 mice. The protective effect is similar to previous studies using transcorneal ES (TcES) to treat the RP animal model, in which both the survival and light response of photoreceptors were improved [9,10,12,13,25,26,27]. Furthermore, we analyzed the light responses of individual RGC, the output neuron that transmit visual signal from the retina to the brain. We found that TsES improved the SNR and light sensitivity of RGCs mainly by decreasing the abnormally high spontaneous firing. Similar improvement of SNR and decrease of spontaneous firing of RGCs was also reported when applying TcES on MNU-injured mice, a rapid photoreceptor degeneration RP model [11]. Therefore, the new strategy of TsES is able to protect degenerated retinas similarly to TcES.

While there were functional improvement of photoreceptors and RGCs, TsES at 100 μA failed to improve the visual ability of rd10 mice to detect luminance (Figure 5). This may be correlated with the reduction of the light response of ON-bipolar cells by TsES (Figure 2B), since the impairment of ON pathway may hinder the animal to tell light from the darkness. Reducing the current of TsES to 50 μA failed to improve the retinal function. It even impaired the function of rd10 RGC, including reducing peak and average light responses and decreasing SNR and light sensitivity. Why different amplitude of TsES acts differently remains unclear, but the current-dependent changes of retinal proteins after TcES may help to explain [28]. A study explored the protein changes in healthy rat eyes after TcES and reported a current strength dependent change in the proteins involved in synaptic transmission and intercellular Ca^2+^ regulation: more proteins were upregulated or downregulated at 100 μA than at 50 μA [28]. Staining for ribbon structure, patch-clamping bipolar cells, and calcium imaging of retinal neurons may be performed in future studies to clarify the current strength-dependent effect.

This indicated that, even with the same pulse frequency and treatment duration, the amplitude of the electric current may influence the ES therapeutic effects. Before long-term application on the degenerating retina, a short-term study is necessary to find a safe and protective current amplitude at certain stage in a specific retinal disease.

### 4.2. TsES Is as Effective as TcES in Protecting the Degenerated Retina

Many parameters of the electric current affect the therapeutic effects, including pulse frequency, amplitude, and treatment duration [29]. Comparing to widely used TcES, the protective threshold of the current used in TsES was similar. In TcES applying biphasic rectangular wave pulses, 50–700 μA was used. In this TsES treatment, current range was narrowed down to 50–100 μA. This effective current of 100 μA was similar to the study of RCS rats [11]. TcES at 100 μA or above were effective to enhance ERG response or photoreceptor survival, but a lower current at 50 μA hardly improved the survival of photoreceptors in RCS rats [9]. To treat transgenic photoreceptor degenerated animals, the duration of TcES treatment was a few weeks (up to 20 weeks) for RCS or P23H-1 rats [9,13,25] and rhodopsin P347L transgenic rabbits [12]. For rd10 mice, we applied TsES for 3–5 days and in another study TcES treatment was applied as short as 5 days [14]. However, in their study, the starting point was chosen at a late stage when all photoreceptors degenerated and focused on the excitation of the cortex instead of the retina. Here, we started the treatment at an early time before rod apoptosis reaches a peak and reported an improvement in retinal function. Therefore, with a minimum current strength and short treatment duration, we provide the proof of concept of applying TsES as a new strategy to protect degenerated retinas with similar efficiency as TcES.

### 4.3. Properties of the Current microERG

In the current study, we repetitively applied a 2 s light flash to induce the responses in retinal neurons recorded by MEA as we previously do [19]. The flash intensity we used (4.20 to 7.7 log photons/μm^2^/s) may sound high enough to bleach the rod and light-adapted the retina during the stimuli, which was indicated by the tiny N1 wave (or a-wave according to other reports). However, we specially chose the intensity range (4.20 to 7.7 log photons/μm^2^/s) to maximum match the dynamic intensity rang of RGC spiking (Figure 4B). Using intensity lower than this can hardly induce any RGC spiking nor microERG response and using higher intensity will not further increase the RGC spiking. Similar intensity of flash was also applied in other work [30,31,32,33,34,35], so despite the possible bleaching of rods, we cannot use any lower flash intensity for the MEA recording in our current setup. Since we are comparing the retinal light response among different treatment groups under the same recording conditions, the bleaching or light-adapted condition will not affect the major conclusion.

The waveforms of microERG recorded by MEA may not equal to those reported in vivo. First, the conditions of microERG recording from isolated retina (ex vivo) by MEA array is very different from those of in vivo ERG recording. For mciroERG, there is no RPE, and the field potential is recorded by small electrode on the array, which only collects local changes instead of whole retina. Second, the waveform is affected by many factors, including the attachment of the retina to each individual electrode, focal damages to the retina during the preparation procedures or degenerating status, and the status of dark or light adaptation after repeated light stimuli [20]. Indeed, labs using different flash intensities and durations obtained different shapes of waveforms [19,20,35,36], and a slow rising positive peak (P2 wave, or c-wave in ERG) was observed as we found here [20,35]. Without RPE which believes to contribute to c-wave, it is highly possible that the P2 wave is not the traditional c-wave in ERG. Therefore, whether the waveforms in microERG are identical to those in traditional in vivo ERG remains uncertain.

Though cellular origin of each peak of ERG recording in vivo is well explained, the origins of peaks in microERG from isolated retina deserves further investigation. By applying pharmacological agents to block ON pathway by L-AP4 and OFF pathways by cocktails of glutamate receptors, Fujii et al. identified the cellular origin of a-wave (i.e., N1 wave in Figure 2) and b-wave (i.e., P1 wave in Figure 2) as photoreceptors and ON bipolar cells respectively in the isolated retina from normal mice [20]. Therefore, they concluded the microERG waveforms recorded by MEA were similar to those in traditional ERG. However, they also found that in rd1 mice when most photoreceptors degenerated, a clear a-wave was still observed in microERG recording and exposure to L-AP abolished both the a- and b-waves [20], which question the contribution of photoreceptors to a-wave. Recently, Kralik et al. (2021) applied L-AP4 on the retina from young rd1 mice that photoreceptors still remained, the negative a-wave was totally eliminated, indicating a contribution of ON bipolar cells to this wave [37]. In our previous study [19], we tried to identify the cellular origin of microERG in our own setup. Adding L-AP4 and then additional NBQX and AP5 in normal mouse retina eliminated P1 wave (which we labelled as a-wave there) and then P2 wave, leaving only N2 wave (which we labelled as b-wave there). Therefore, we concluded that during the sustained light stimuli, ON bipolar cells contribute to the P1 wave, OFF bipolar cells contribute to the P2 wave and photoreceptors contribute to the N2 wave, though N2 wave is suggested to arise from Muller glia in traditional ERG recording [38]. As N3 and P3 waves were induced after light offset, we think they may arise from OFF bipolar cells, which is consistent with in vivo ERG.

### 4.4. Limitation of the Current Study

With the proof of concept that TsES application protected the degenerating retina, our study has a few limitations. The major limitation is that the improvement of photoreceptor survival is subtle though significant. A further study applying a stronger current than 100 μA or with a longer treatment duration than 30 min may help to improve the protective effect of TsES. Starting the treatment at earlier time of retinal degeneration may also help. To observe the effectiveness of TsES on the visual function in cortex, we may also need to extend the treatment period.

The major mechanisms explaining protective effect of ES in eye diseases include inhibiting neuronal apoptosis and microglial activation, upregulating neurotrophic factors in Muller cells, enhancing retinal blood flow, and modulating brain plasticity [18]. Though we would expect a similar mechanism as TcES, our current study focused on reporting the treatment effect without going further to explore the underlying mechanism of TsES. Further studies collecting retinal tissues after treatment and analyzing the above-mentioned pathways are needed in the future studies.

Furthermore, as ES can directly modulate ion channels, it may be worth to apply TsES on animal models of retinal dystrophies related to ion channel mutation besides rd10 mouse. Many gene mutations in ocular ion channels were reported (e.g., *CACAN1A*, *CACNG8*, *CNGB3*) to contribute to retinal dystrophy. A deep transcriptomic analysis further suggests a complex regulation of these ion channel-related mutated genes on neurotransmission associated to light stimuli [39], therefore future study may test the efficacy of TsES on these animal models and explore how ES modulate the underlying ion channel related pathways.

## 5. Conclusions

Our current study provided a new strategy for applying ES on the sclera instead of the cornea to treat retinal degeneration to avoid cornea injury. TsES may provide a new long-term strategy to delay the retinal photoreceptor degeneration.

## Data Availability

Data will be available upon request.

## References

[1] Hamel C. (2006). Retinitis pigmentosa. Orphanet J. Rare Dis..

[2] Verbakel S.K., van Huet R.A.C., Boon C.J.F., den Hollander A.I., Collin R.W.J., Klaver C.C.W., Hoyng C.B., Roepman R., Klevering B.J. (2018). Non-syndromic retinitis pigmentosa. Prog. Retin. Eye Res..

[3] Gallenga C.E., Lonardi M., Pacetti S., Violanti S.S., Tassinari P., Di Virgilio F., Tognon M., Perri P. (2021). Molecular Mechanisms Related to Oxidative Stress in Retinitis Pigmentosa. Antioxid.

[4] Kaur G., Singh N.K. (2021). The Role of Inflammation in Retinal Neurodegeneration and Degenerative Diseases. Int. J. Mol. Sci..

[5] Lang M., Harris A., Ciulla T.A., Siesky B., Patel P., Belamkar A., Mathew S., Verticchio Vercellin A.C. (2019). Vascular dysfunction in retinitis pigmentosa. Acta Ophthalmol..

[6] Wang A.L., Knight D.K., Vu T.T., Mehta M.C. (2019). Retinitis Pigmentosa: Review of Current Treatment. Int. Ophthalmol. Clin..

[7] Zhao S., Mehta A.S., Zhao M. (2020). Biomedical applications of electrical stimulation. Cell. Mol. Life Sci..

[8] Liu J., Tong K., Lin Y., Lee V.W.H., So K.F., Shih K.C., Lai J.S.M., Chiu K. (2021). Effectiveness of Microcurrent Stimulation in Preserving Retinal Function of Blind Leading Retinal Degeneration and Optic Neuropathy: A Systematic Review. Neuromodulation.

[9] Morimoto T., Fujikado T., Choi J.S., Kanda H., Miyoshi T., Fukuda Y., Tano Y. (2007). Transcorneal electrical stimulation promotes the survival of photoreceptors and preserves retinal function in royal college of surgeons rats. Investig. Ophthalmol. Vis. Sci..

[10] Ni Y.Q., Gan D.K., Xu H.D., Xu G.Z., Da C.D. (2009). Neuroprotective effect of transcorneal electrical stimulation on light-induced photoreceptor degeneration. Exp. Neurol..

[11] Tao Y., Chen T., Liu Z.Y., Wang L.Q., Xu W.W., Qin L.M., Peng G.H., Yi-Fei H. (2016). Topographic Quantification of the Transcorneal Electrical Stimulation (TES)-Induced Protective Effects on N-Methyl-N-Nitrosourea-Treated Retinas. Investig. Ophthalmol. Vis. Sci..

[12] Morimoto T., Kanda H., Kondo M., Terasaki H., Nishida K., Fujikado T. (2012). Transcorneal electrical stimulation promotes survival of photoreceptors and improves retinal function in rhodopsin P347L transgenic rabbits. Investig. Ophthalmol. Vis. Sci..

[13] Rahmani S., Bogdanowicz L., Thomas J., Hetling J.R. (2013). Chronic delivery of low-level exogenous current preserves retinal function in pigmented P23H rat. Vision Res..

[14] Agadagba S.K., Li X., Chan L.L.H. (2020). Excitation of the Pre-frontal and Primary Visual Cortex in Response to Transcorneal Electrical Stimulation in Retinal Degeneration Mice. Front. Neurosci..

[15] Kurimoto T., Ueda K., Mori S., Sakamoto M., Yamada-Nakanishi Y., Matsumiya W., Nakamura M. (2019). A study protocol for evaluating the efficacy and safety of skin electrical stimulation for Leber hereditary optic neuropathy: A single-arm, open-label, non-randomized prospective exploratory study. Clin. Ophthalmol..

[16] Barhoum R., Martinez-Navarrete G., Corrochano S., Germain F., Fernandez-Sanchez L., de la Rosa E.J., de la Villa P., Cuenca N. (2008). Functional and structural modifications during retinal degeneration in the rd10 mouse. Neuroscience.

[17] He L.M., Sun Z.Q., Li J.S., Zhu R., Niu B., Tam K.L., Xiao Q., Li J., Wang W.J., Tsui C.Y. (2021). Electrical stimulation at nanoscale topography boosts neural stem cell neurogenesis through the enhancement of autophagy signaling. Biomaterials.

[18] Liu J., Ma A.K.H., So K.F., Lee V.W.H., Chiu K. (2022). Mechanisms of electrical stimulation in eye diseases: A narrative review. Adv. Ophthalmol. Pract. Res..

[19] Liu F., Liu X.B., Zhou Y.M., Yu Y.K., Wang K., Zhou Z.Q., Gao H., So K.F., Vardi N., Xu Y. (2021). Wolfberry-derived zeaxanthin dipalmitate delays retinal degeneration in a mouse model of retinitis pigmentosa through modulating STAT3, CCL2 and MAPK pathways. J. Neurochem..

[20] Fujii M., Sunagawa G.A., Kondo M., Takahashi M., Mandai M. (2016). Evaluation of micro Electroretinograms Recorded with Multiple Electrode Array to Assess Focal Retinal Function. Sci. Rep..

[21] Barrett J.M., Degenaar P., Sernagor E. (2015). Blockade of pathological retinal ganglion cell hyperactivity improves optogenetically evoked light responses in rd1 mice. Front. Cell. Neurosci..

[22] Toychiev A.H., Ivanova E., Yee C.W., Sagdullaev B.T. (2013). Block of gap junctions eliminates aberrant activity and restores light responses during retinal degeneration. J. Neurosci..

[23] Tao Y., Chen T., Liu B., Xue J.H., Zhang L., Xia F., Pang J.J., Zhang Z.M. (2013). Visual signal pathway reorganization in the Cacna1f mutant rat model. Investig. Ophthalmol. Vis. Sci..

[24] Pu M., Xu L., Zhang H. (2006). Visual response properties of retinal ganglion cells in the royal college of surgeons dystrophic rat. Investig. Ophthalmol. Vis. Sci..

[25] Hanif A.M., Kim M.K., Thomas J.G., Ciavatta V.T., Chrenek M., Hetling J.R., Pardue M.T. (2016). Whole-eye electrical stimulation therapy preserves visual function and structure in P23H-1 rats. Exp. Eye Res..

[26] Schatz A., Arango-Gonzalez B., Fischer D., Enderle H., Bolz S., Rock T., Naycheva L., Grimm C., Messias A., Zrenner E. (2012). Transcorneal Electrical Stimulation Shows Neuroprotective Effects in Retinas of Light-Exposed Rats. Investig. Ophthalmol. Vis. Sci..

[27] Tao Y., Chen T., Liu B., Wang L.Q., Peng G.H., Qin L.M., Yan Z.J., Huang Y.F. (2016). The transcorneal electrical stimulation as a novel therapeutic strategy against retinal and optic neuropathy: A review of experimental and clinical trials. Int. J. Ophthalmol.

[28] Kanamoto T., Souchelnytskyi N., Kurimoto T., Ikeda Y., Sakaue H., Munemasa Y., Kiuchi Y. (2015). Proteomic Study of Retinal Proteins Associated with Transcorneal Electric Stimulation in Rats. J. Ophthalmol..

[29] Morimoto T., Miyoshi T., Sawai H., Fujikado T. (2010). Optimal parameters of transcorneal electrical stimulation (TES) to be neuroprotective of axotomized RGCs in adult rats. Exp. Eye Res..

[30] Alarautalahti V., Ragauskas S., Hakkarainen J.J., Uusitalo-Jarvinen H., Uusitalo H., Hyttinen J., Kalesnykas G., Nymark S. (2019). Viability of Mouse Retinal Explant Cultures Assessed by Preservation of Functionality and Morphology. Investig. Ophthalmol. Vis. Sci..

[31] Gauvain G., Akolkar H., Chaffiol A., Arcizet F., Khoei M.A., Desrosiers M., Jaillard C., Caplette R., Marre O., Bertin S. (2021). Optogenetic therapy: High spatiotemporal resolution and pattern discrimination compatible with vision restoration in non-human primates. Commun. Biol..

[32] Kralik J., van Wyk M., Stocker N., Kleinlogel S. (2022). Bipolar cell targeted optogenetic gene therapy restores parallel retinal signaling and high-level vision in the degenerated retina. Commun. Biol..

[33] Lagali P.S., Balya D., Awatramani G.B., Münch T.A., Kim D.S., Busskamp V., Cepko C.L., Roska B. (2008). Light-activated channels targeted to ON bipolar cells restore visual function in retinal degeneration. Nat. Neurosci..

[34] Laprell L., Tochitsky I., Kaur K., Manookin M.B., Stein M., Barber D.M., Schön C., Michalakis S., Biel M., Kramer R.H. (2017). Photopharmacological control of bipolar cells restores visual function in blind mice. J. Clin. Investig..

[35] Hughes S., Rodgers J., Hickey D., Foster R.G., Peirson S.N., Hankins M.W. (2016). Characterisation of light responses in the retina of mice lacking principle components of rod, cone and melanopsin phototransduction signalling pathways. Sci. Rep..

[36] Kireev D., Montes V.R., Stevanovic J., Srikantharajah K., Offenhausser A. (2019). N(3)-MEA Probes: Scooping Neuronal Networks. Front. Neurosci..

[37] Kralik J., Kleinlogel S. (2021). Functional Availability of ON-Bipolar Cells in the Degenerated Retina: Timing and Longevity of an Optogenetic Gene Therapy. Int. J. Mol. Sci..

[38] Heikkinen H., Vinberg F., Pitkanen M., Kommonen B., Koskelainen A. (2012). Flash responses of mouse rod photoreceptors in the isolated retina and corneal electroretinogram: Comparison of gain and kinetics. Investig. Ophthalmol. Vis. Sci..

[39] Donato L., Scimone C., Alibrandi S., Abdalla E.M., Nabil K.M., D’Angelo R., Sidoti A. (2020). New Omics-Derived Perspectives on Retinal Dystrophies: Could Ion Channels-Encoding or Related Genes Act as Modifier of Pathological Phenotype?. Int. J. Mol. Sci..

